# Targeting focal adhesions:*Helicobacter pylori*-host communication in cell migration

**DOI:** 10.1186/1478-811X-6-2

**Published:** 2008-08-06

**Authors:** Sabine Schneider, Christiane Weydig, Silja Wessler

**Affiliations:** 1 Junior Research Group, Paul-Ehrlich Institut, D-63225 Langen, Germany

## Abstract

Highly dynamic integrin-based focal adhesions provide an important structural basis for anchoring the cellular actin cytoskeleton to the surrounding extracellular matrix. The human pathogen *Helicobacter pylori *(*H. pylori*) directly targets integrins with drastic consequences on the epithelial cell morphology and migration, which might contribute to the disruption of the gastric epithelium *in vivo*. In this review, we summarize the recent findings concerning the complex mechanism through which *H. pylori *interferes with host integrin signaling thereby deregulating focal adhesions and the actin cytoskeleton of motile epithelial cells.

## Review

The human pathogen *H. pylori *colonizes the stomachs of more than 50% of the world's population. The molecular interaction between *H. pylori *and cells of the gastric epithelium is thought to be the major factor inducing inflammatory responses of the infected host, which can result in the development of the malignant diseases gastric cancer or lymphoma of the MALT (mucosa-associated lymphoid tissue) system [1]. The pathogenesis of *H. pylori *mainly depends on the exposure of several bacterial factors, including cytotoxin-associated gene A (CagA), the type IV secretion system (T4SS), vacuolating cytotoxin A (VacA), outer inflammatory protein A (OipA) and several adherence factors, to the host [2-4]. Due to their pivotal role in *H. pylori *pathogenesis, these factors are currently being intensively studied to elucidate how they induce specific host responses.

Much interest has been focused on the pathogenic factor CagA, which is transported via the T4SS into the host cytoplasm where it activates signal transduction pathways leading to cancer-associated processes [5]. Once injected into the host cytosol, the Glu-Pro-Ile-Tyr-Ala sequences (EPIYA motifs) in the CagA protein (CagA^PY^) are successively phosphorylated by non-receptor tyrosine kinases of the Src and Abl families [6,7]. A direct causal link between CagA^PY ^and *in vivo *oncogenesis has recently been demonstrated in transgenic mice expressing CagA. These mice developed gastric polyps and adenocarcinoma of the stomach and small intestine [8]. However, the detailed molecular mechanism of CagA^PY ^action in infected cells is not completely understood, although the injected CagA might imitate eukaryotic adaptor proteins by recruiting host signaling factors into protein complexes, both in a phosphorylation-dependent and a phosphorylation-independent manner [9-11]. Indeed, by generating a transgenic *Drosophila *model, it has been demonstrated that CagA expression rescues photoreceptor development in the absence of the *Drosophila *Grb2-associated binder (Gab) homolog Daughter of Sevenless (DOS) indicating that CagA can mimic the function of a Gab adaptor protein within the *Drosophila *tissue [12]. Hence, it is not surprising that the number of CagA-interacting proteins is steadily increasing. These proteins are involved in diverse cellular signal transduction pathways targeting cell proliferation, cellular junctions and adhesions [9-11].

**Figure 1:**
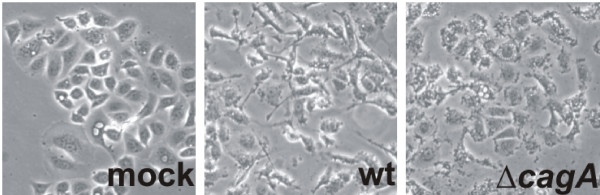
**The *H. pylori*-mediated hummingbird phenotype involves cell elongation and migration**. While non-infected gastric epithelial AGS cells (mock) show a round morphology, infection with *H. pylori *wild type (wt) induced loss of cell-to-cell contacts, cell elongation and migration. The elongated cell morphology in response to *H. pylori *is dependent on the injection of CagA, since AGS cells infected with a *cagA*-deficient *H. pylori *mutant (Δ*cagA*) do not elongate.

A hallmark of cultured *H. pylori*-infected epithelial cells is the development of the so-called hummingbird phenotype, the characteristic formation of which is dependent on CagA^PY ^(Fig. 1) [13]. This phenotype might influence the immune response, wound healing, metastasis or invasive growth of cancer cells *in vivo *[14,15]. The *H. pylori*-induced hummingbird phenotype is reminiscent of growth factor-induced cell scattering, which consists of several processes, namely (i) cell movement, driven by rearrangement of the cytoskeleton and (ii) the assembly/disassembly of cell-matrix contacts [16]. However, the way in which *H. pylori *regulates these cellular processes is even less understood. Here, we review the recent progress in the study of *H. pylori *communication with intercellular signal transduction pathways controlling the coordinated action of cytoskeletal-dependent migration, cell morphology and cell-matrix adhesion, all of which might contribute to the pathogenesis of *H. pylori*.

### Cellular aspects of cell migration

Cell migration comprises several temporally and spatially coordinated events, including elongation of the leading edge, adhesion of this protrusion to the matrix, movement of the cell body and release of the trailing edge of the cell [14,15]. Important structures are focal adhesions (FAs) and the actin cytoskeleton, which are strictly regulated during cell migration.

FAs are comprised of α and β integrin heterodimers that form a bridge between the intracellular actin cytoskeleton and the extracellular matrix (ECM) [17]. While the extracellular domain of integrins binds directly to ECM proteins, the cytoplasmic tail is linked to the actin cytoskeleton via a steadily increasing number of signaling and adapter proteins, such as focal adhesion kinase (FAK), vinculin, talin and paxillin. [17]. Initially, integrins were presumed to act simply as cell adhesion receptors, but it has become clear that they also play crucial roles in the communication of cellular signal transduction pathways leading to adhesion and rearrangement of the actin cytoskeleton [14,18]. The integrity and stability of proteins located in the FA site are mainly regulated by tyrosine phosphorylation, which is controlled by classical "outside in" and "inside out" signaling cascades [19].

**Figure 2:**
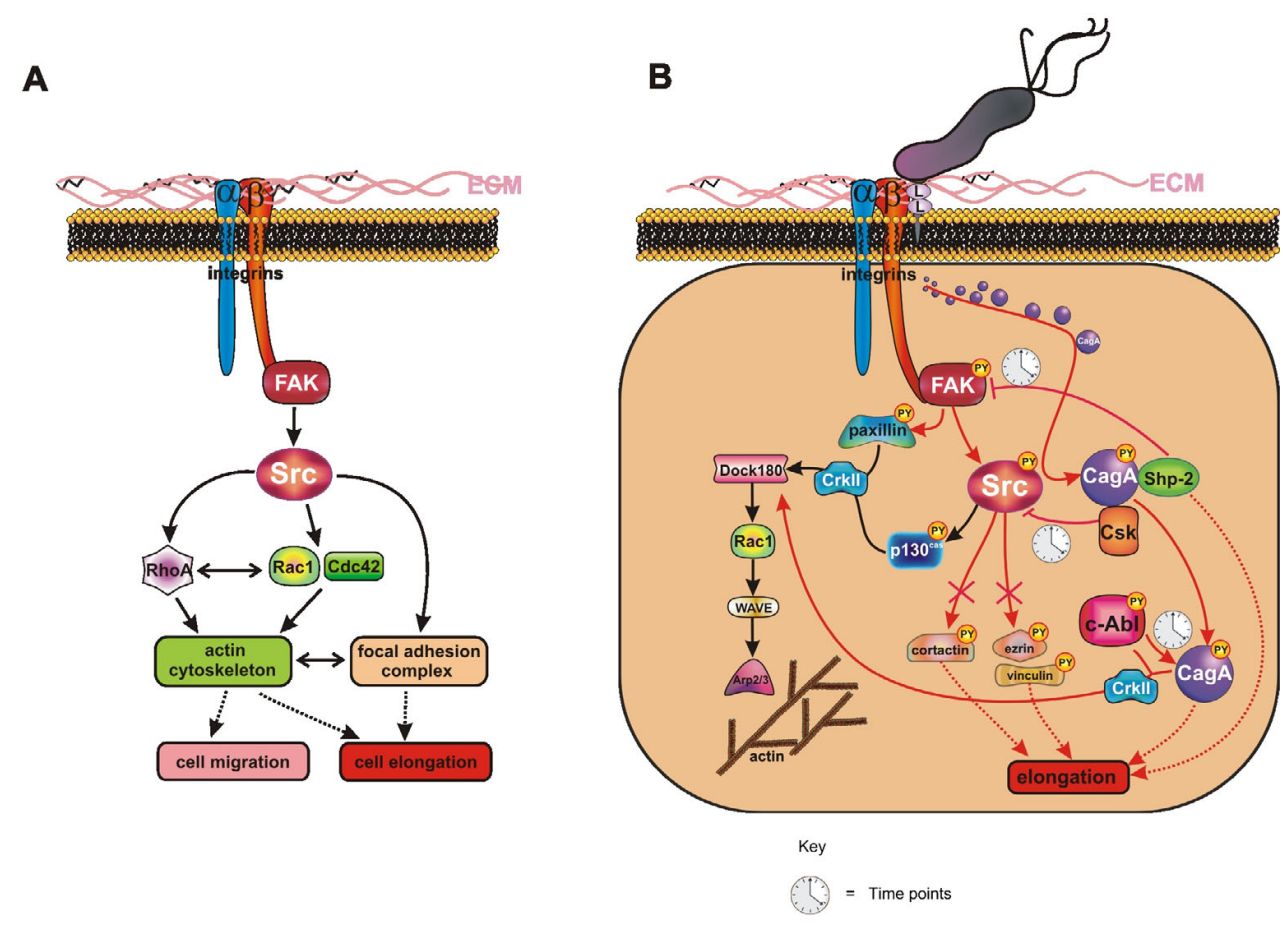
**Schematic overview of the integrin signaling**. **(A) Signaling pathways downstream of FAK and Src controlling actin cytoskeletal rearrangement and FAs**. Integrin activation induces recruitment and stimulation of FAK and Src, which target RhoA or Rac1 and Cdc42 GTPases, thereby controlling cell migration and elongation via regulation of the actin cytoskeleton. The elongated cell morphology might also be caused by deregulated FAs. **(B) Detailed mechanism of CagL-induced integrin signaling leading to cell elongation and reorganization of the actin cytoskeleton**. FAK and Src are activated via integrin-CagL (L) interaction leading to the injection of the pathogenic factor CagA. CagA is initially phosphorylated by Src, which then interacts with Shp-2 and Csk to inactivate FAK and Src at later time points. Inactivation of Src leads to the dephosphorylation of ezrin, vinculin and cortactin, while CagA tyrosine phosphorylation is maintained by activated Abl kinases. These processes contribute to the deregulation of FA disassembly leading to host cell elongation. In parallel, FAK and Src control the actin cytoskeleton via the CrkII/DOCK180/Rac1/WAVE/Arp2/3 pathway, which affects cell migration. This pathway is controlled by Abl kinases, paxillin or p130Cas. Red arrowheads:*H. pylori*-associated signaling pathways; black arrowheads: FAK- and Src-associated signaling pathways.

However, cell migration does not only require a coordinated assembly and disassembly of FAs to move the cell body on the ECM, but also needs active actin polymerization along the plasma membrane to contract the cell cortex. The Rho family of small GTPases, including the well studied members Cdc42, Rac1 and RhoA, is a key regulator of the cytoskeleton (Fig. 2A). Generation of cortical tension and a rounded cell morphology are critically controlled by FAK, Src and the small GTPase RhoA, while Rac1 and Cdc42 direct actin assembly to generate lamellipodia and elongation [20,21].

Besides Rho-GTPase-controlled actin rearrangements, cell morphology might also be influenced by deregulated FA-based cell adhesion resulting in the development of high traction forces on both cell poles and in a drastic cell elongation (Fig. 2A). Indeed, video microscopy has revealed that cells infected with *H. pylori *fail to release their back ends during cell migration, probably through the maintenance of vinculin-containing FA complexes at their distal tips [22]. This observation indicates that *H. pylori *causes a FA-dependent retraction defect of motile cells leading to the elongated cell morphology, the molecular mechanism of which will be discussed below.

### *H. pylori *injects CagA across FAs and regulates the elongation of infected epithelial cells

Like the growth factor-induced scattering phenotype, the hummingbird phenotype involves an altered cell morphology and migration, which are regulated by *H. pylori*. Injection of CagA is strongly associated with the development of an elongated cell morphology, while the process of migration requires a functional T4SS system, but not the CagA protein itself [23-25]. These observations led to the speculation that either another *H. pylori *factor translocates through the T4SS into host cells or that the T4SS pilus directly interacts with an unknown cell surface receptor to stimulate specific signal transduction pathways leading to the motogenic response of *H. pylori*-colonized epithelial cells [26]. Indeed, peptidoglycan has been shown to translocate into the host cytoplasm via the T4SS pilus, where it binds to the nucleotide-binding oligomerization domain-containing 1 (Nod1) protein leading to the transactivation of nuclear factor kappa B (NF-κB)-dependent proinflammatory genes [27]. Whether this factor also plays a role in the rearrangement of the actin cytoskeleton has not yet been investigated.

The existence of a T4SS receptor on the host cell surface has recently been demonstrated. It was shown that CagA injection requires binding of the bacterial adhesin CagL, located on the tip of the T4SS, to the β1 integrin receptor of epithelial cells (Fig. 2B) [28]. In earlier studies, CagL was already found to be essential for the injection of the CagA protein and the induction of IL-8 secretion [26,29]. Similar to other integrin-binding ECM proteins, *H. pylori *CagL possesses a specific Arg-Gly-Asp (RGD) motif, which mediates the binding of CagL to integrin α5β1 [28]. The biological importance of integrins for *H. pylori *infection has been further emphasized by the finding that β2 integrins are VacA receptors on T lymphocytes, allowing *H. pylori *to subvert the host immune response [30]. However, VacA can also interact with the ECM protein fibronectin *in vitro*, and this interaction can be partly inhibited by RGD-containing peptides [31]. Thus, it is not completely clear whether the interaction of VacA and β2 integrin is direct or requires fibronectin. In epithelial cells, the CagL-α5β1 interaction allows translocation of CagA into the host cytoplasm and also concomitantly activates the integrin-dependent tyrosine phosphorylation of CagA at FA sites [28]. In this and another recent study, it was further shown that activated β1 integrins are also required for CagA-independent signaling pathways involved cell migration (Fig. 2B) [32].

The detailed mechanism of how CagA is injected after integrin binding is not known, while the mechanism of integrin-triggered CagA phosphorylation is better understood. The intracellular signaling initiated by integrins in FAs is mainly mediated via FAK and Src kinases (Fig. 2A) [33]. The clustering of integrins leads to the rapid recruitment of FAK to the FA complex, where it is autophosphorylated on tyrosine 397 (Y397) [34]. This leads to the recruitment and activation of Src family kinases, which, together with FAK, are central in the regulation of downstream signaling pathways that control cell spreading, cell movement and cell survival [34,35]. Phosphorylation of FAK at Y397 correlates with increased catalytic activity and appears to be important for the tyrosine phosphorylation of focal complex-associated proteins such as paxillin. In agreement with this, both FAK and Src have been shown to be activated by the CagL-α5β1 interaction within the first 120 minutes of *H. pylori *infection [28], which then leads to the phosphorylation of paxillin (Fig. 2B) [32]. The interplay between *H. pylori *and integrins has been confirmed in a study by Tabassam and colleagues, with the major difference that these authors postulate that the outer membrane protein OipA of *H. pylori *is important for FAK and Src activation [36]. Hence, it might be relevant to determine whether OipA interaction with the host cell membrane mediates a tight interaction between *H. pylori *and host cells, allowing CagL to interact with β1 integrin, or whether OipA also targets β1 integrins directly. *H. pylori*-activated Src has been shown to directly phosphorylate EPIYA motifs of injected CagA, which serves as a signal to form a complex composed of CagA^PY ^and Src homology 2 domain-containing tyrosine phosphatase (Shp-2) *in vitro *as well as *in vivo *(Fig. 2B) [37,38]. The interaction of CagA^PY ^and Shp-2 is important in the induction of the elongated cell morphology [39-41], potentially through the *H. pylori*-activated Shp-2/Rap1/B-Raf/Erk signaling pathway [42,43].

However, FAK and Src are rapidly inactivated in cells that have been infected with *H. pylori *for extended time points [44-47]. Inactivation of FAK and Src is mediated via different molecular mechanisms (Fig. 2B). In transfection studies, binding of CagA^PY ^to Shp-2 induced an increased phosphatase activity of Shp-2, resulting in a direct dephosphorylation and inactivation of FAK [47]. Another mechanism has been postulated for the inactivation of Src, involving inhibition by a negative feedback-loop mechanism initiated by CagA^PY^, which directly interacts with the C-terminal Src kinase (Csk) to inactivate Src activity [46]. Although Src inactivation leads to the dephosphorylation of other Src substrates, such as vinculin, cortactin and ezrin [45,48,49], the tyrosine phosphorylation of CagA is maintained by Abl kinases in late phase infections [6,7], ensuring that CagA^PY ^constitutively stimulates signaling pathways in host cells (Fig. 2B). Altogether, sustained CagA phosphorylation by Abl kinases, and the inactivated proteins FAK, Src with their dephosphorylated substrates have been demonstrated to be relevant signaling elements in cell elongation, even though their detailed functional roles in FAs and the corresponding molecular mechanisms are not clear and should be investigated in future studies.

### *H. pylori *regulates the actin cytoskeleton of infected epithelial cells

The CagL-integrin-mediated CagA injection is crucial for deregulation of FAs; however, rearrangement of the actin cytoskeleton leading to cell migration appears to be mainly independent of CagA, but requires a functional T4SS [23-25,50]. In particular, *H. pylori *mutants that do not express the CagL protein are unable to stimulate cell migration, while *H. pylori *strains that are deficient for CagA still activate motility to a certain extent [23]. This led to the hypothesis that CagL-mediated stimulation of the β1 integrin/FAK/Src pathway is involved in the cytoskeletal rearrangement.

Actin cytoskeleton dynamics are regulated by Rho family GTPases [51]. In *H. pylori*-infected cells, the Rho GTPase Rac1, but not RhoA or Cdc42, has been shown to be a crucial component of the CagA-induced phenotype [52]. A major function of activated Rac1 is to stimulate actin polymerization via WAVE (WASP family verprolin-homologous protein) and the Arp2/3 (actin-related protein 2/3) complex, leading to plasma membrane protrusion and extension of lamellipodia [53]. FAK/Src signaling in particular has been implicated in the regulation of Rac1 activity through two well characterized downstream pathways involving the scaffolding proteins p130Cas and paxillin (Fig. 2B), which are both enriched in FAs [51]. Upon phosphorylation by Src, p130Cas can recruit a Crk (v-crk sarcoma virus CT10 oncogene homolog)/DOCK180 (dedicator of cytokinesis) complex that has GEF (guanine nucleotide exchange factor) activity toward Rac1 [54,55]. The critical role of Crk adaptor proteins in the actin cytoskeleton rearrangement of *H. pylori*-infected cells has recently been demonstrated [52,56]. Moreover, the Crk/DOCK180/Rac1/WAVE/Arp2/3 signal transduction pathway is stimulated in *H. pylori*-infected cells [56], indicating that recruitment of p130Cas into the Crk/DOCK180 complex might be an important event regulating the Rac1-dependent actin cytoskeleton responses and plasma membrane protrusion of *H. pylori*-infected cells (Fig. 2B). On the other hand, *H. pylori*-targeted FAK phosphorylates paxillin [32], which might also contribute to the activity of the Crk/DOCK180 complex (Fig. 2B), but which additionally suppresses RhoA [51]. As RhoA can inhibit Rac1, this pathway could also be important for efficient integrin-stimulated activation of Rac1 [51]. Taken together, these studies reveal diverse mechanisms through which FAK/Src signaling coordinates Rac1 activation, thereby controlling the actin cytoskeleton in *H. pylori*-infected cells (Fig. 2B).

The actin cytoskeletal rearrangement might also be influenced by the Abl-dependent signaling pathways. In contrast to FAK and Src [28], Abl kinases remain active in *H. pylori*-infected cells [6,7]. Since the upstream signal transduction pathway leading to sustained Abl kinase activity has not yet been investigated, it is tempting to speculate whether the kinase activity of c-Abl is dependent on CagL-integrin signaling or on a physical interaction with CagA^PY^, as has been observed upon *H. pylori *infection [6]. Interestingly, CagA^PY ^binds directly to the adapter proteins of the Crk family [56], which have also been found to be directly tyrosine-phosphorylated by *H. pylori*-activated Abl [6,7]. The complex composed of Abl, CagA^PY ^and CrkII might then activate the DOCK180/Rac1/WAVE/Arp2/3 pathway leading to the actin cytoskeletal rearrangement (Fig. 2B) [56].

### Conclusion: Does *H. pylori *interfere with FA maturation *in vivo*?

**Figure 3:**
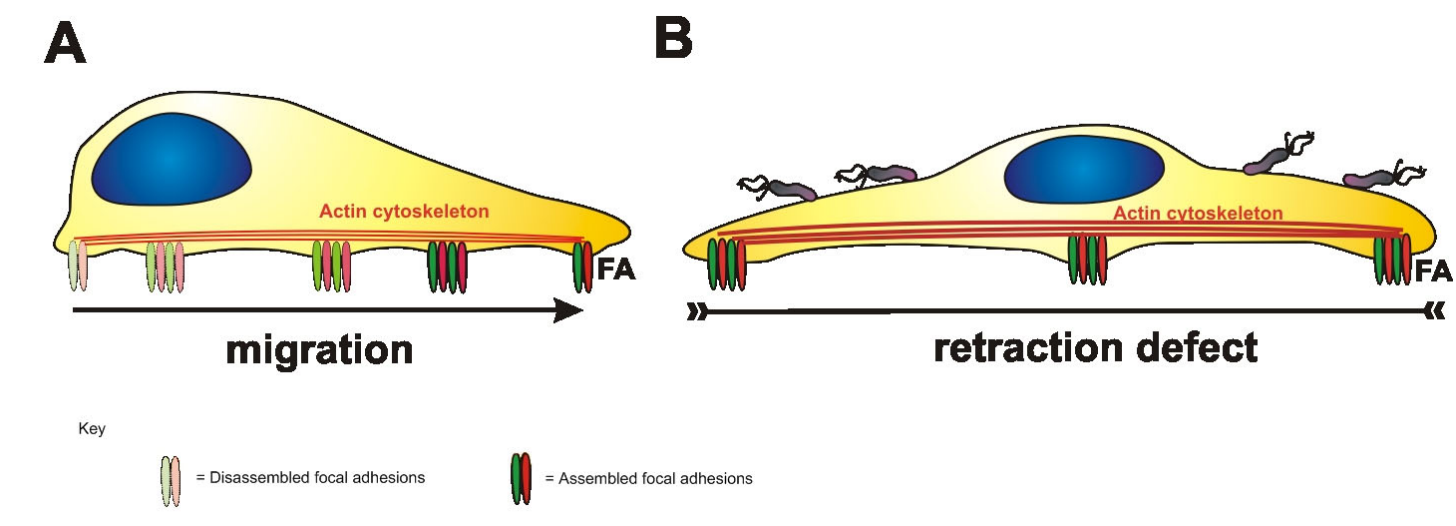
**Model of *H. pylori*-dependent cell elongation**. Cells require a coordinated action of the actin cytoskeleton and FAs to migrate efficiently. **(A) **New FAs are assembled at the leading edge of motile cells, which are then disassembled after maturation at the site of retraction. **(B) **Motile cells infected with *H. pylori *become markedly elongated. This involves CagA-independent cytoskeletal rearrangements and a CagA-dependent retraction defect, which probably acts by stabilizing maturing FAs.

Drastic cell elongation and migration are hallmarks of *H. pylori*-infected epithelial cells *in vitro*. Motile cells need to assemble new FAs at the leading edge, but require the disassembly of these structures at the trailing edge to move the cell body efficiently (Fig. 3A). *H. pylori *clearly deregulates host integrin-dependent signal transduction pathways, leading to the generation of cell tension, elongation and migration through synchronous processes. While migration of *H. pylori*-infected cells is driven by rearrangements of the actin cytoskeleton, motile cells fail to release their back ends during cell locomotion. Hence, it is necessary to ask whether *H. pylori *interferes with the maturation of FAs to alter the epithelial morphology (Fig. 3B). There is little published data concerning the regulation of trailing and maturating FAs and how turnover is regulated in motile cells. However, the idea that FAs represent the crucial gate for injection of *H. pylori *CagA into the host cytoplasm is striking and opens a new field for investigating the mechanism through which CagA^PY ^interferes with the stability and maturation of FAs *in vitro *as well as *in vivo*.

FAs were initially identified in cultured fibroblasts and for a long time it was speculated that they were artificially formed structures in cultured cells. Today, it is well established that FAs exist *in vivo*, mediating cell-matrix junctions at the basolateral surface of polarized cells [57]. The fact that CagA injection requires integrin activation leads to the question of whether *H. pylori *can contact FAs at the basolateral membrane *in vivo*. Several studies have shown that CagA is necessary for the disruption of the intercellular tight junctions of polarized cells [58-60]. Furthermore, it has been demonstrated that injected CagA preferentially localizes to the apical membrane of T84 cells [61]. Detailed models have been proposed in recent reviews [11,62], suggesting that moderate amounts of CagA might be injected across the apical membrane without the need of integrins in early phases of infection. Together with other secreted bacterial and/or host factors (e.g. soluble *H. pylori *factors or cellular matrix metalloproteases), injected CagA then supports the disruption of intercellular adhesions [25,63,64] allowing *H. pylori *to enter the intercellular space. In fact, in biopsies from patients with intestinal metaplasia and gastric cancer, *H. pylori *was found in the intercellular space and lamina propria [65], indicating that *H. pylori *has access to FAs at later time points of infection *in vivo*. Consequently, it is important to analyze the consequences of CagA injection and FA deregulation *in vivo*. CagA^PY^-associated cell elongation possibly hinders *H. pylori*-mediated cell migration, since failure of tail retraction might result in a reduced migration speed. In contrast, elongation allows cells to pass tissues more easily, which would support invasive growth of single cells. Therefore, analyzing the consequences of cell elongation and migration *in vivo *should lead to novel insights into *H. pylori *pathogenesis.

## Competing interests

The authors declare that they have no competing interests.

## Authors' contributions

SS and SW drafted and wrote the manuscript. CW participated in the design of the manuscript and prepared the figures. All authors read and approved the final manuscript.

## References

[B1] Blaser MJ, Atherton JC (2004). *Helicobacter pylori *persistence: biology and disease. J Clin Invest.

[B2] Clyne M, Dolan B, Reeves EP (2007). Bacterial factors that mediate colonization of the stomach and virulence of Helicobacter pylori. FEMS Microbiol Lett.

[B3] Backert S, Meyer TF (2006). Type IV secretion systems and their effectors in bacterial pathogenesis. Curr Opin Microbiol.

[B4] Rieder G, Fischer W, Haas R (2005). Interaction of *Helicobacter pylori* with host cells: function of secreted and translocated molecules. Curr Opin Microbiol.

[B5] Backert S, Feller SM, Wessler S (2008). Emerging roles of Abl family tyrosine kinases in microbial pathogenesis. Trends Biochem Sci.

[B6] Poppe M, Feller SM, Romer G, Wessler S (2007). Phosphorylation of *Helicobacter pylori* CagA by c-Abl leads to cell motility. Oncogene.

[B7] Tammer I, Brandt S, Hartig R, Konig W, Backert S (2007). Activation of Abl by Helicobacter pylori: a novel kinase for CagA and crucial mediator of host cell scattering. Gastroenterology.

[B8] Ohnishi N, Yuasa H, Tanaka S, Sawa H, Miura M, Matsui A, Higashi H, Musashi M, Iwabuchi K, Suzuki M, Yamada G, Azuma T, Hatakeyama M (2008). Transgenic expression of *Helicobacter pylori* CagA induces gastrointestinal and hematopoietic neoplasms in mouse. Proc Natl Acad Sci U S A.

[B9] Hatakeyama M (2003). *Helicobacter pylori* CagA--a potential bacterial oncoprotein that functionally mimics the mammalian Gab family of adaptor proteins. Microbes Infect.

[B10] Backert S, Selbach M (2005). Tyrosine-phosphorylated bacterial effector proteins: the enemies within. Trends Microbiol.

[B11] Wessler S, Backert S (2008). Molecular mechanisms of epithelial-barrier disruption by Helicobacter pylori. Trends Microbiol.

[B12] Botham CM, Wandler AM, Guillemin K (2008). A transgenic *Drosophila * model demonstrates that the *Helicobacter pylori* CagA protein functions as a eukaryotic Gab adaptor. PLoS Pathog.

[B13] Asahi M, Azuma T, Ito S, Ito Y, Suto H, Nagai Y, Tsubokawa M, Tohyama Y, Maeda S, Omata M, Suzuki T, Sasakawa C (2000). *Helicobacter pylori* CagA protein can be tyrosine phosphorylated in gastric epithelial cells. J Exp Med.

[B14] Ridley AJ, Schwartz MA, Burridge K, Firtel RA, Ginsberg MH, Borisy G, Parsons JT, Horwitz AR (2003). Cell migration: integrating signals from front to back. Science.

[B15] Broussard JA, Webb DJ, Kaverina I (2008). Asymmetric focal adhesion disassembly in motile cells. Curr Opin Cell Biol.

[B16] Boyer B, Valles AM, Edme N (2000). Induction and regulation of epithelial-mesenchymal transitions. Biochem Pharmacol.

[B17] Lo SH (2006). Focal adhesions: what's new inside. Dev Biol.

[B18] Hynes RO (2002). Integrins: bidirectional, allosteric signaling machines. Cell.

[B19] Lock JG, Wehrle-Haller B, Stromblad S (2008). Cell-matrix adhesion complexes: master control machinery of cell migration. Semin Cancer Biol.

[B20] Ridley AJ (2006). Rho GTPases and actin dynamics in membrane protrusions and vesicle trafficking. Trends Cell Biol.

[B21] Sahai E, Marshall CJ (2003). Differing modes of tumour cell invasion have distinct requirements for Rho/ROCK signalling and extracellular proteolysis. Nat Cell Biol.

[B22] Bourzac KM, Botham CM, Guillemin K (2007). *Helicobacter pylori* CagA Induces AGS Cell Elongation through a Cell Retraction Defect That Is Independent of Cdc42, Rac1, and Arp2/3. Infect Immun.

[B23] Al Ghoul L, Wessler S, Hundertmark T, Kruger S, Fischer W, Wunder C, Haas R, Roessner A, Naumann M (2004). Analysis of the type IV secretion system-dependent cell motility of Helicobacter pylori-infected epithelial cells. Biochem Biophys Res Commun.

[B24] Moese S, Selbach M, Kwok T, Brinkmann V, Konig W, Meyer TF, Backert S (2004). *Helicobacter pylori* induces AGS cell motility and elongation via independent signaling pathways. Infect Immun.

[B25] Weydig C, Starzinski-Powitz A, Carra G, Lower J, Wessler S (2007). CagA-independent disruption of adherence junction complexes involves E-cadherin shedding and implies multiple steps in *Helicobacter pylori* pathogenicity. Exp Cell Res.

[B26] Fischer W, Puls J, Buhrdorf R, Gebert B, Odenbreit S, Haas R (2001). Systematic mutagenesis of the *Helicobacter pylori* cag pathogenicity island: essential genes for CagA translocation in host cells and induction of interleukin-8. Mol Microbiol.

[B27] Viala J, Chaput C, Boneca IG, Cardona A, Girardin SE, Moran AP, Athman R, Memet S, Huerre MR, Coyle AJ, DiStefano PS, Sansonetti PJ, Labigne A, Bertin J, Philpott DJ, Ferrero RL (2004). Nod1 responds to peptidoglycan delivered by the *Helicobacter pylori* cag pathogenicity island. Nat Immunol.

[B28] Kwok T, Zabler D, Urman S, Rohde M, Hartig R, Wessler S, Misselwitz R, Berger J, Sewald N, Konig W, Backert S (2007). *Helicobacter* exploits integrin for type IV secretion and kinase activation. Nature.

[B29] Censini S, Lange C, Xiang Z, Crabtree JE, Ghiara P, Borodovsky M, Rappuoli R, Covacci A (1996). cag, a pathogenicity island of Helicobacter pylori, encodes type I-specific and disease-associated virulence factors. Proc Natl Acad Sci U S A.

[B30] Sewald X, Gebert-Vogl B, Prassl S, Barwig I, Weiss E, Fabbri M, Osicka R, Schiemann M, Busch DH, Semmrich M, Holzmann B, Sebo P, Haas R (2008). Integrin subunit CD18 Is the T-lymphocyte receptor for the *Helicobacter pylori* vacuolating cytotoxin. Cell Host Microbe.

[B31] Hennig EE, Godlewski MM, Butruk E, Ostrowski J (2005). *Helicobacter pylori* VacA cytotoxin interacts with fibronectin and alters HeLa cell adhesion and cytoskeletal organization in vitro. FEMS Immunol Med Microbiol.

[B32] Snider JL, Allison C, Bellaire BH, Ferrero RL, Cardelli JA (2008). The {beta}1 Integrin Activates JNK Independent of CagA, and JNK Activation Is Required for *Helicobacter pylori* CagA+-induced Motility of Gastric Cancer Cells. J Biol Chem.

[B33] Mitra SK, Schlaepfer DD (2006). Integrin-regulated FAK-Src signaling in normal and cancer cells. Curr Opin Cell Biol.

[B34] Parsons JT, Martin KH, Slack JK, Taylor JM, Weed SA (2000). Focal adhesion kinase: a regulator of focal adhesion dynamics and cell movement. Oncogene.

[B35] Caron-Lormier G, Berry H (2005). Amplification and oscillations in the FAK/Src kinase system during integrin signaling. J Theor Biol.

[B36] Tabassam FH, Graham DY, Yamaoka Y (2008). OipA plays a role in Helicobacter pylori-induced focal adhesion kinase activation and cytoskeletal re-organization. Cell Microbiol.

[B37] Higashi H, Tsutsumi R, Muto S, Sugiyama T, Azuma T, Asaka M, Hatakeyama M (2002). SHP-2 tyrosine phosphatase as an intracellular target of *Helicobacter pylori* CagA protein. Science.

[B38] Yamazaki S, Yamakawa A, Ito Y, Ohtani M, Higashi H, Hatakeyama M, Azuma T (2003). The CagA protein of *Helicobacter pylori* is translocated into epithelial cells and binds to SHP-2 in human gastric mucosa. J Infect Dis.

[B39] Backert S, Moese S, Selbach M, Brinkmann V, Meyer TF (2001). Phosphorylation of tyrosine 972 of the *Helicobacter pylori* CagA protein is essential for induction of a scattering phenotype in gastric epithelial cells. Mol Microbiol.

[B40] Selbach M, Moese S, Hauck CR, Meyer TF, Backert S (2002). Src is the kinase of the *Helicobacter pylori* CagA protein in vitro and in vivo. J Biol Chem.

[B41] Stein M, Bagnoli F, Halenbeck R, Rappuoli R, Fantl WJ, Covacci A (2002). c-Src/Lyn kinases activate *Helicobacter pylori* CagA through tyrosine phosphorylation of the EPIYA motifs. Mol Microbiol.

[B42] Higashi H, Nakaya A, Tsutsumi R, Yokoyama K, Fujii Y, Ishikawa S, Higuchi M, Takahashi A, Kurashima Y, Teishikata Y, Tanaka S, Azuma T, Hatakeyama M (2004). *Helicobacter pylori* CagA induces Ras-independent morphogenetic response through SHP-2 recruitment and activation. J Biol Chem.

[B43] Wessler S, Rapp UR, Wiedenmann B, Meyer TF, Schoneberg T, Hocker M, Naumann M (2002). B-Raf/Rap1 signaling, but not c-Raf-1/Ras, induces the histidine decarboxylase promoter in *Helicobacter pylori* infection. FASEB J.

[B44] Pai R, Cover TL, Tarnawski AS (1999). *Helicobacter pylori* vacuolating cytotoxin (VacA) disorganizes the cytoskeletal architecture of gastric epithelial cells. Biochem Biophys Res Commun.

[B45] Selbach M, Moese S, Hurwitz R, Hauck CR, Meyer TF, Backert S (2003). The *Helicobacter pylori* CagA protein induces cortactin dephosphorylation and actin rearrangement by c-Src inactivation. EMBO J.

[B46] Tsutsumi R, Higashi H, Higuchi M, Okada M, Hatakeyama M (2003). Attenuation of *Helicobacter pylori* CagA x SHP-2 signaling by interaction between CagA and C-terminal Src kinase. J Biol Chem.

[B47] Tsutsumi R, Takahashi A, Azuma T, Higashi H, Hatakeyama M (2006). Focal adhesion kinase is a substrate and downstream effector of SHP-2 complexed with *Helicobacter pylori* CagA. Mol Cell Biol.

[B48] Moese S, Selbach M, Brinkmann V, Karlas A, Haimovich B, Backert S, Meyer TF (2007). The *Helicobacter pylori* CagA protein disrupts matrix adhesion of gastric epithelial cells by dephosphorylation of vinculin. Cell Microbiol.

[B49] Selbach M, Moese S, Backert S, Jungblut PR, Meyer TF (2004). The *Helicobacter pylori* CagA protein induces tyrosine dephosphorylation of ezrin. Proteomics.

[B50] Churin Y, Kardalinou E, Meyer TF, Naumann M (2001). Pathogenicity island-dependent activation of Rho GTPases Rac1 and Cdc42 in *Helicobacter pylori* infection. Mol Microbiol.

[B51] Siesser PM, Hanks SK (2006). The signaling and biological implications of FAK overexpression in cancer. Clin Cancer Res.

[B52] Brandt S, Shafikhani S, Balachandran P, Jin S, Hartig R, Konig W, Engel J, Backert S (2007). Use of a novel coinfection system reveals a role for Rac1, H-Ras, and CrkII phosphorylation in Helicobacter pylori-induced host cell actin cytoskeletal rearrangements. FEMS Immunol Med Microbiol.

[B53] Cory GO, Ridley AJ (2002). Cell motility: braking WAVEs. Nature.

[B54] Kiyokawa E, Hashimoto Y, Kobayashi S, Sugimura H, Kurata T, Matsuda M (1998). Activation of Rac1 by a Crk SH3-binding protein, DOCK180. Genes Dev.

[B55] Gu J, Sumida Y, Sanzen N, Sekiguchi K (2001). Laminin-10/11 and Fibronectin Differentially Regulate Integrin- dependent Rho and Rac Activation via p130Cas-CrkII-DOCK180 Pathway. J Biol Chem.

[B56] Suzuki M, Mimuro H, Suzuki T, Park M, Yamamoto T, Sasakawa C (2005). Interaction of CagA with Crk plays an important role in Helicobacter pylori-induced loss of gastric epithelial cell adhesion. J Exp Med.

[B57] Fuchs E, Dowling J, Segre J, Lo SH, Yu QC (1997). Integrators of epidermal growth and differentiation: distinct functions for beta 1 and beta 4 integrins. Curr Opin Genet Dev.

[B58] Amieva MR, Vogelmann R, Covacci A, Tompkins LS, Nelson WJ, Falkow S (2003). Disruption of the epithelial apical-junctional complex by *Helicobacter pylori* CagA. Science.

[B59] Bagnoli F, Buti L, Tompkins L, Covacci A, Amieva MR (2005). *Helicobacter pylori* CagA induces a transition from polarized to invasive phenotypes in MDCK cells. Proc Natl Acad Sci U S A.

[B60] Saadat I, Higashi H, Obuse C, Umeda M, Murata-Kamiya N, Saito Y, Lu H, Ohnishi N, Azuma T, Suzuki A, Ohno S, Hatakeyama M (2007). *Helicobacter pylori* CagA targets PAR1/MARK kinase to disrupt epithelial cell polarity. Nature.

[B61] El Etr SH, Mueller A, Tompkins LS, Falkow S, Merrell DS (2004). Phosphorylation-independent effects of CagA during interaction between *Helicobacter pylori* and T84 polarized monolayers. J Infect Dis.

[B62] Mimuro H, Berg DE, Sasakawa C (2008). Control of epithelial cell structure and developmental fate: lessons from Helicobacter pylori. Bioessays.

[B63] Lytton SD, Fischer W, Nagel W, Haas R, Beck FX (2005). Production of ammonium by *Helicobacter pylori* mediates occludin processing and disruption of tight junctions in Caco-2 cells. Microbiology.

[B64] Papini E, Satin B, Norais N, de BM, Telford JL, Rappuoli R, Montecucco C (1998). Selective increase of the permeability of polarized epithelial cell monolayers by *Helicobacter pylori* vacuolating toxin. J Clin Invest.

[B65] Necchi V, Candusso ME, Tava F, Luinetti O, Ventura U, Fiocca R, Ricci V, Solcia E (2007). Intracellular, intercellular, and stromal invasion of gastric mucosa, preneoplastic lesions, and cancer by Helicobacter pylori. Gastroenterology.

